# Preoperative Cannabis Use and Ankle ORIF Outcomes: Higher Risks of Infection, Nonunion, and Reoperation

**DOI:** 10.1177/10711007251385971

**Published:** 2025-11-16

**Authors:** Sri Tummala, Brandon A. Wood, Mehul M. Mittal, Senthil N. Sambandam, Dane K. Wukich

**Affiliations:** 1 Department of Orthopaedic Surgery, University of Texas Southwestern Medical Center, Dallas, TX, USA; 2 Texas A&M College of Medicine, Dallas, TX, USA; 3 University of Texas Southwestern Medical School, Dallas, TX, USA; 4 Department of Prosthetics and Orthotics, University of Texas Southwestern Medical Center, Dallas, TX, USA

**Keywords:** Cannabis, Ankle Fracture, Open Reduction Internal Fixation, ORIF, Nonunion, Reoperation, Outcomes, Risk Factors

## Abstract

**Background:**

With the widespread legalization and rising prevalence of cannabis use, understanding its impact on perioperative outcomes has become clinically critical. Although recent orthopaedic research has examined cannabis-related risks following various procedures, its implications following surgical fixation of ankle fractures remain unexplored. Given the increasing incidence and complexity of ankle fractures, clarifying these risks is essential for effective perioperative management and patient counseling.

**Methods:**

A retrospective cohort analysis was conducted using a nationally representative research database. Patients undergoing open reduction and internal fixation (ORIF) for rotational ankle fractures were identified using standardized *Current Procedural Terminology* (*CPT*) codes and stratified by preoperative active cannabis use. Propensity score matching (PSM) was used to control for 27 demographic and clinical confounders; data were drawn from the TriNetX Research Network; postmatch balance is reported. Outcomes assessed included medical complications and health care utilization at 90 days and 6 months, and surgical outcomes at 3 years postoperatively. Relative risks (RRs) with 95% confidence intervals were calculated. To account for multiple testing across 34 outcomes, a Bonferroni correction was applied, with statistical significance defined as *P* < .0015.

**Results:**

After PSM, 3126 matched pairs were included in the analysis. At 90 days postoperatively, cannabis use was significantly associated with increased risks of postoperative infection (RR = 1.696, 95% CI 1.230-2.337, *P* < .001), wound dehiscence (RR = 1.781, 95% CI 1.268-2.502, *P* < .001), and inpatient readmissions (RR = 1.724, 95% CI 1.221-2.435, *P* < .001). These associations persisted at 6 months, including elevated risks of postoperative infection (RR = 1.745, 95% CI 1.304-2.337, *P* < .001), sepsis (RR = 2.847, 95% CI 1.477-5.488, *P* < .001), and hardware infection (RR = 1.944, 95% CI 1.291-2.929, *P* < .001). At 3 years, the cannabis-use cohort was associated with significantly increased risks of nonunion (RR = 2.308, 95% CI 1.207-4.413, *P* < .001) and reoperation (RR = 2.168, 95% CI 1.353-3.475, *P* < .001). No significant differences were observed for other measured outcomes following Bonferroni adjustment.

**Conclusion:**

Preoperative cannabis use is associated with significantly increased complications following ankle ORIF, notably systemic and hardware infections, nonunion, and reoperation. These findings support recognizing cannabis use as a potentially modifiable perioperative risk factor, akin to tobacco use, underscoring the importance of targeted counseling and strategies aimed at optimizing surgical and patient outcomes.

## Graphical abstract

**Graphical Abstract:**
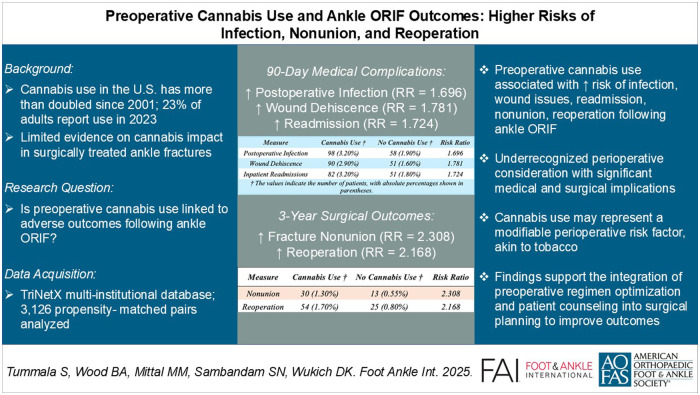
This is a visual representation of the abstract.

**Level of Evidence:** Level III, case control-study or retrospective cohort study.

## Introduction

Ankle fractures represent a substantial and increasing public health concern in developed nations, currently occurring at an incidence of approximately 150 per 100 000 individuals annually.^13,30,31^ The surgical management of these fractures may be complicated by postoperative issues such as infections, delayed wound healing, and implant-related issues.^1,8,22^ Identifying and managing modifiable perioperative risk factors is essential for improving clinical outcomes in patients undergoing surgical treatment for ankle fractures.

Among known risk factors, smoking has been consistently linked with increased complication rates following ankle fracture surgeries, notably manifesting as prolonged postoperative pain, persistent swelling, surgical site infections, delayed wound healing, and both nonunion and malunion complications.^3,12,17^ Because of these risks, surgeons routinely emphasize smoking cessation as a critical component of perioperative optimization strategies for foot and ankle conditions.^9^

Concurrently with the increase in surgically treated ankle fractures, there has been a notable rise in the prevalence of cannabis use, driven partly by its popularity as an analgesic alternative amid the ongoing opioid crisis. Medical cannabis use was legalized in 34 US states by March 2021, with an additional 15 states permitting recreational use, reflecting widespread societal acceptance and expanding clinical application.^4,11^ Between 2001 and 2013, cannabis use in the United States more than doubled from 4.1% to 9.5%, mirroring a global usage prevalence of approximately 3.8%.^4,11^ Particularly among young adults, cannabis use surged significantly from 29% in 2011 to approximately 43% by 2021.^18^

Despite the growing body of research exploring the perioperative implications of cannabis use across various surgical procedures, specific and comprehensive investigations in the context of ankle fractures remain notably sparse.^6,29^ Given the evolving legal landscape and rapidly increasing cannabis consumption, this study seeks to specifically evaluate the impact of preoperative cannabis use on short-term medical complications and health care use, as well as long-term outcomes such as nonunion and reoperation rates following surgical management of rotational ankle fractures. Through this evaluation, the study aims to provide critical insights for evidence-based preoperative risk stratification, targeted patient counseling, and potential optimization of cannabis use as a modifiable perioperative risk factor analogous to tobacco smoking. The authors hypothesized that preoperative cannabis use will be associated with increased rates of medical and surgical complications following ankle fracture ORIF.

## Materials and Methods

### Data Source

The data for this study were obtained from the TriNetX Research Network, which compiles electronic medical records from more than 150 million patients across 94 health care organizations. This comprehensive data set includes demographics, medication histories, diagnostic records, procedural details, and laboratory results. All patient information is deidentified in full compliance with the Health Insurance Portability and Accountability Act (HIPAA), ensuring that individually identifiable information is neither collected, used, nor transmitted. Because of the deidentified nature of the data set, the current research was exempt from institutional review board approval.

### Study Design

On March 10, 2025, a query was performed within the TriNetX Research Network using coding systems including *International Classification of Diseases, Tenth Revision* (*ICD-10*), *Current Procedural Terminology* (*CPT*), and Veteran Affairs Formulary (VA) codes. Patients eligible for inclusion underwent open reduction and internal fixation (ORIF) for rotational ankle fractures specifically involving medial malleolar fractures (*CPT* 27766), lateral malleolar fractures (*CPT* 27792), posterior malleolar fractures (*CPT* 27769), bimalleolar fractures (*CPT* 27814), trimalleolar fractures (*CPT* 27822, 27823), or syndesmotic disruption (*CPT* 27829).

The identified patients were subsequently classified into 2 distinct cohorts based on their preoperative cannabis use status. Cannabis usage was identified using *ICD-9* and *ICD-10* codes for cannabis use, abuse, or dependence (*ICD-9*: 304.30, 305.20; *ICD-10*: F12.10, F12.20, F12.90). Patients who had documented cannabis use within 3 months before surgery were assigned to the cannabis-use study group, whereas patients without documented cannabis usage in the same preoperative period were placed into the control group (no cannabis use). To improve specificity in identifying active cannabis users, patients in the cannabis cohort were required to have at least 1 cannabis-related *ICD-9* or *ICD-10* diagnosis recorded within 1 year before surgery and a second instance of the same diagnosis within 3 months prior to surgery. This dual time-point requirement was intended to capture sustained or active cannabis use rather than a single, possibly remote, diagnosis.

**Figure 1.**
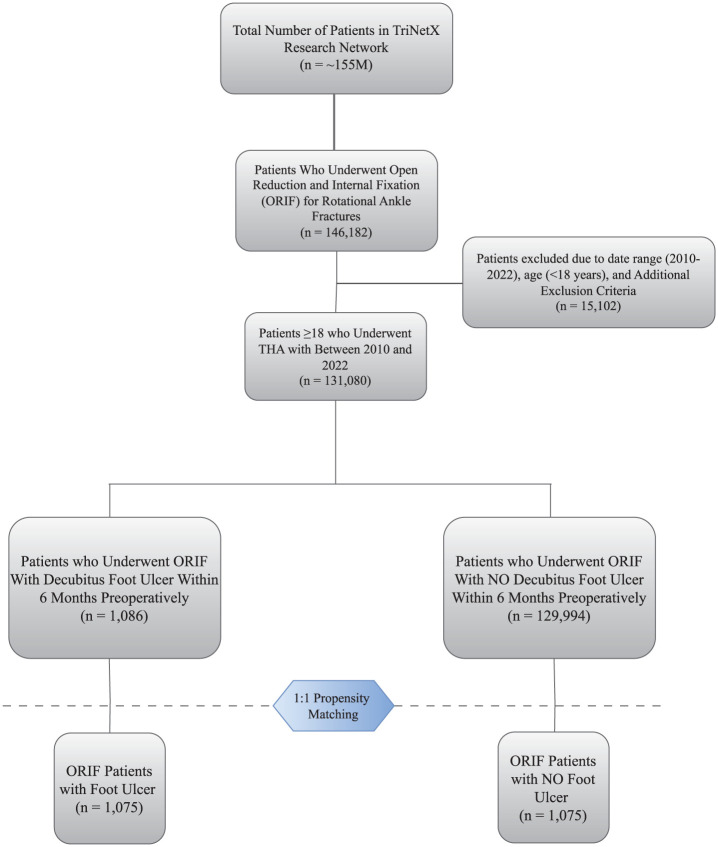
STROBE flowchart for patient selection.

The date of surgery was defined as the index date for both cohorts. This retrospective cohort study adhered to the Strengthening the Reporting of Observational Studies in Epidemiology (STROBE) guidelines, with detailed patient selection criteria illustrated in Figure 1.

### Outcomes Evaluated

Outcomes were assessed at multiple postoperative intervals. Medical complications evaluated at 90 days and 6 months postoperatively included postoperative infection, sepsis, wound disruption, cellulitis, deep vein thrombosis, pulmonary embolism, urinary tract infection, opioid usage, acute renal failure, pneumonia, emergency department visits, and inpatient readmissions.

Long-term surgical outcomes assessed at 3 years postoperatively included nonunion, malunion, hardware infection, reoperation procedures, and ankle arthroplasty. Additionally, below-knee amputation was assessed at 5 years postoperatively. All *ICD-10* codes used to identify evaluated outcomes are detailed in the Appendix B.

### Propensity-Score Matching

Propensity score matching is a statistical approach employed to mitigate bias in observational research by pairing subjects who share similar covariate profiles. In this study, we initially conducted regression analysis to identify covariates significantly associated with both the exposure and the primary outcomes. Variables incorporated in propensity score matching were age at index date, sex, race, body mass index (BMI, categorized), alcohol abuse (*ICD-10*: F10), tobacco use (*ICD-10*: Z72.0), diabetes mellitus (*ICD-10*: E08-E13), chronic kidney disease (*ICD-10*: N18), disorders of bone mineral density (*ICD-10*: M80-M85), liver disease (*ICD-10*: K70-K77), essential hypertension (*ICD-10*: I10), ischemic heart disease (*ICD-10*: I20-I25), chronic obstructive pulmonary disease (*ICD-10*: J44), history of slipping/tripping/stumbling/falling (*ICD-10*: W00-W19), hyperlipidemia (*ICD-10*: E78.5), chronic glucocorticoid use (VA: HS051), persons with potential health hazards related to socioeconomic and psychosocial circumstances (*ICD-10*: Z55-Z64), and long-term nonsteroidal anti-inflammatory drug use (CN104). By pairing patients with closely matched propensity scores, we aimed to achieve covariate balance, thereby minimizing confounding. Matching was performed using a greedy nearest-neighbor algorithm.

Matching quality was verified by ensuring the standardized mean differences were below 0.1 for all matching variables, indicating successful balancing. Comprehensive comparison data of baseline characteristics before and after matching are presented in Appendix B.

### Statistical Analysis

Statistical analyses included calculation of both absolute risk differences and relative risks (RRs), each with corresponding 95% confidence intervals. Fisher exact test, χ² test, and Student *t* test were used for categorical and continuous variables, respectively. These measures were reported for outcomes meeting or approaching significance to provide clinically meaningful context alongside relative effect sizes. To account for multiple comparisons across postoperative time points, we applied a Bonferroni correction. A total of 34 outcomes were evaluated (14 at 90 days, 14 at 6 months, and 6 across the 3- and 5-year follow-up periods). The Bonferroni-adjusted significance threshold was calculated as 0.05 ÷ 34 = 0.00147. Accordingly, statistical significance was defined as *p* <.0015. All analyses were performed using R software (Vienna, Austria) and the analytical tools within the TriNetX platform.

## Results

### Patient Demographics and Characteristics Analysis

Before propensity score matching, a total of 3140 patients in the cannabis use cohort and 122 338 patients in the non–cannabis use cohort were identified. Baseline characteristics for both cohorts before and after matching are detailed in Appendix B.

Before matching, patients in the cannabis use cohort were significantly younger (mean age: 37.6 vs 46.6 years; *P* < .001), and a higher proportion were Black or African American (27.1% vs 14.2%; *P* < .001). This cohort also had higher rates of alcohol abuse (29.0% vs 5.9%; *P* < .001), tobacco use (11.7% vs 3.1%; *P* < .001), chronic glucocorticoid use (40.4% vs 31.3%; *P* < .001), long-term nonsteroidal anti-inflammatory drug use (40.5% vs 25.4%; *P* < .001), and documented socioeconomic and psychosocial hardship (11.2% vs 2.5%; *P* < .001).

Following matching, 3126 cannabis-using patients were matched to an equal number of non–cannabis-using patients. Postmatch analysis demonstrated successful covariate balancing across all variables, with standardized mean differences less than 0.1 for all matched characteristics, indicating minimal residual bias between cohorts (Appendix B).

### Ninety-Day Medical Complications and Health Care Utilization

At 90 days postoperatively, the cannabis use cohort exhibited significantly higher rates of postoperative infection (3.20% vs 1.90%, *P* < .001), wound dehiscence (2.90% vs 1.60%, *P* < .001), and inpatient readmissions (3.20% vs 1.80%, *P* < .001) compared with the non–cannabis use cohort. Sepsis (0.90% vs 0.30%, *P* = .003) and hardware infection (1.90% vs 1.00%, *P* = .005) approached significance but did not meet the Bonferroni-adjusted threshold. No significant differences were observed in mortality, deep vein thrombosis, pulmonary embolism, urinary tract infection, opioid use, acute renal failure, pneumonia, cellulitis, or emergency department visits (Table 1).

**Table 1.** Medical Outcomes of Cannabis Use vs Nonusers at 90 Days: Matched.

Measure	Cannabis Use^a^	No Cannabis Use^a^	Risk Ratio	95% CI	*p* Value^b^
Medical complications					
Mortality	≤10^c^	≤10^c^	–	–	–
Postoperative infection	98 (3.20)	58 (1.90)	1.696	(1.230, 2.337)	**<.001**
Sepsis	28 (0.90)	10 (0.30)	2.814	(1.369, 5.783)	.003
Wound dehiscence	90 (2.90)	51 (1.60)	1.781	(1.268, 2.502)	**<.001**
Cellulitis	68 (2.40)	44 (1.50)	1.587	(1.090, 2.310)	.015
Deep vein thrombosis	26 (0.80)	24 (0.80)	1.084	(0.624, 1.884)	.774
Pulmonary embolism	12 (0.40)	11 (0.40)	1.096	(0.484, 2.480)	.826
Urinary tract infection	25 (0.90)	23 (0.80)	1.103	(0.628, 1.939)	.733
Opioid use	11 (13.40)	19 (11.00)	1.221	(0.610, 2.446)	.573
Acute renal failure	17 (0.60)	18 (0.60)	0.977	(0.504, 1.892)	.944
Pneumonia	12 (0.50)	14 (0.60)	0.862	(0.399, 1.859)	.704
Hardware infection	46 (1.90)	23 (1.00)	2.003	(1.218, 3.294)	.005
Health care utilization					
Inpatient readmissions	82 (3.20)	51 (1.80)	1.724	(1.221, 2.435)	**<.001**
Emergency department visits	29 (3.40)	38 (3.80)	0.894	(0.556, 1.437)	.643

a The values indicate the number of patients, with percentages shown in parentheses.

b Significant values are bolded (*p* < .0015).

c Patient counts of ≤10 are not reported on TriNetX.

### Six-Month Medical Complications and Health care Utilization

At 6 months postoperatively, the cannabis use cohort demonstrated significantly higher rates of postoperative infection (3.90% vs 2.20%, *P* < .001), sepsis (1.10% vs 0.40%, *P* < .001), hardware infection (2.80% vs 1.40%, *P* < .001), and inpatient readmissions (3.40% vs 2.00%, *P* < .001) compared with patients without cannabis use. Wound dehiscence (3.20% vs *P* = .004) and cellulitis (3.40% vs 2.10%, *P* = .004) approached significance but did not meet the Bonferroni-adjusted threshold. No statistically significant differences were observed between cohorts in mortality, deep vein thrombosis, pulmonary embolism, urinary tract infection, opioid use, acute renal failure, pneumonia, or emergency department visits (Table 2).

**Table 2.** Medical Outcomes of Cannabis Use vs Nonusers at 6 Months: Matched.

Measure	Cannabis Use^a^	No Cannabis Use^a^	Risk Ratio	95% CI	*p* Value^b^
Medical complications					
Mortality	14 (0.40)	≤10^c^	–	–	–
Postoperative infection	120 (3.90)	69 (2.20)	1.745	(1.304, 2.337)	**<.001**
Sepsis	34 (1.10)	12 (0.40)	2.847	(1.477, 5.488)	**<.001**
Wound dehiscence	97 (3.20)	62 (2.00)	1.579	(1.153, 2.164)	.004
Cellulitis	94 (3.40)	61 (2.10)	1.582	(1.151, 2.175)	.004
Deep vein thrombosis	29 (0.90)	28 (0.90)	1.037	(0.618, 1.738)	.891
Pulmonary embolism	18 (0.60)	15 (0.50)	1.205	(0.609, 2.388)	.591
Urinary tract infection	31 (1.10)	31 (1.10)	1.015	(0.618, 1.665)	.954
Opioid use	13 (15.90)	20 (11.60)	1.371	(0.718, 2.619)	.340
Acute renal failure	29 (1.00)	26 (0.90)	1.154	(0.681, 1.954)	.595
Pneumonia	27 (0.90)	19 (0.60)	1.437	(0.801, 2.578)	.222
Hardware infection	66 (2.80)	34 (1.40)	1.944	(1.291, 2.929)	**<.001**
Health care utilization					
Inpatient readmissions	88 (3.40)	55 (2.00)	1.716	(1.230, 2.393)	**<.001**
Emergency department visits	41 (4.70)	45 (4.40)	1.067	(0.706, 1.613)	.758

a The values indicate the number of patients, with percentages shown in parentheses.

b Significant values are bolded (*p* < .0015).

c Patient counts of ≤10 are not reported on TriNetX.

### Three-Year Surgical and Mechanical Outcomes

At 3 years postoperatively, cannabis users demonstrated significantly higher rates of nonunion (1.30% vs 0.55%, *P* < .001) and reoperation (1.70% vs 0.80%, *P* < .001) compared with non–cannabis users. Hardware infection (3.80% vs 2.50%, *P* = .004) approached significance but did not meet the Bonferroni-adjusted threshold. No significant differences were observed between cohorts in malunion, ankle arthroplasty, or below-knee amputation at 5 years (Table 3).

**Table 3.** Long-term Surgical Outcomes of Cannabis Use vs Nonusers: Matched.

Measure	Cannabis Use^a^	No Cannabis Use^a^	Risk Ratio	95% CI	*p* Value^b^
3-Year Surgical Outcomes					
Nonunion	30 (1.30)	13 (0.55)	2.308	(1.207, 4.413)	**<.001**
Malunion	18 (0.60)	16 (0.50)	1.122	(0.417, 2.401)	.737
Reoperation	54 (1.70)	25 (0.80)	2.168	(1.353, 3.475)	**<.001**
Hardware infection	119 (3.80)	79 (2.50)	1.511	(1.142, 2.000)	.004
Ankle arthroplasty	20 (0.60)	17 (0.50)	1.176	(0.617, 2.240)	.622
5-y Below-Knee Amputation					
Amputation	13 (0.42)	12 (0.38)	1.083	(0.495, 2.370)	.842

a The values indicate the number of patients, with percentages shown in parentheses.

b Significant values are bolded (*p* < .0015).

To ensure comprehensive reporting, surgical outcomes were also evaluated at earlier intervals. At 1 year postoperatively, cannabis users exhibited significantly increased risks for hardware infection (3.54% vs 2.17%, *P* < .001) and reoperation (1.49% vs 0.66%, *P* = .002), although nonunion was not significantly elevated (0.83% vs 0.66%, *P* = .458). At 2 years, hardware infection (3.84% vs 2.34%, *P* < .001) and reoperation (1.76% vs 0.79%, *P* < .001) remained significantly higher, whereas nonunion continued to show an elevated but nonsignificant risk (0.96% vs 0.73%, *P* = .328).

## Discussion

The present study found preoperative cannabis use to be associated with an increased risk for short- and long-term complications following surgical management of rotational ankle fractures. Our findings characterize a plausible trajectory of adverse outcomes, progressing from early infectious complications to structural compromise and elevated reoperation requirements. Through an approach that assessed outcomes at 90 days, 6 months, and 3 years postoperatively, this study underscores the sustained impact of cannabis use on surgical recovery.

Our data demonstrate a significantly greater frequency of postoperative infections among cannabis users at both 90 days (RR = 1.696) and 6 months (RR = 1.745) following surgery (*P* < .001). The observed association may result from cannabinoid-mediated immunosuppression, specifically Δ9-tetrahydrocannabinol (THC) inhibition of neutrophil chemotaxis, macrophage activation, and proinflammatory cytokine production.^5,6,16^ Such immunomodulatory effects critically impair primary host defenses against pathogens during the critical wound-healing phase, thereby potentiating infection risk. Correspondingly, rates of cellulitis and sepsis were also elevated in cannabis users within 90 days and 6 months, respectively. This further indicates that compromised systemic immunity may facilitate progression from localized infection to systemic illness. These findings align with Ding et al’s^6^ investigation of knee and hip arthroplasty, which reported significantly elevated postoperative infection odds among patients with cannabis use disorder.

Additionally, wound dehiscence demonstrated significantly elevated incidence among cannabis users (RR = 1.781 at 90 days, *P* < .001). Mechanistically, this complication corresponds with established evidence of cannabinoid-mediated modulation of inflammatory pathways during wound healing. Preclinical models indicate that perturbation of CB1 and CB2 receptor signaling dysregulates cytokine cascades, including elevated TNF-α and MCP-1, potentially delaying wound closure and impairing tissue repair.^24^ The ankle's relatively limited soft tissue coverage heightens vulnerability to these effects, where delayed healing and dehiscence substantially increase risks of subsequent hardware exposure and secondary infection. These findings are clinically consequential because wound complications represent prevalent adverse events after ankle fracture fixation, with reported incidence ranging from 5% to 18% depending on injury and patient factors.^10,15,20,26^

Hardware infection rates were significantly higher at 6 months and approached significant at 90 days and 3 years. Hardware infections constitute a particularly challenging complication in orthopaedic trauma because of the inherent challenge of eradicating biofilm-forming pathogens from implant surfaces. Biofilm development facilitates rapid bacterial adherence to biomaterials, evasion of innate and adaptive immune responses, and resistance to antimicrobial agents. This pathognomonic behavior precipitates chronic infections that typically necessitate hardware removal, substantially increasing patient morbidity and reoperation risks.^2,14,23^ The protracted infection risk profile observed suggests a persistent infectious process, which may be potentiated by sustained immunosuppression, superficial wound compromise, and delayed clinical recognition, collectively elevating reoperation probability.

Crucially, nonunion demonstrated significantly elevated prevalence among cannabis users at 3 years postoperatively, with more than twice the risk observed (RR = 2.308, *P* < .001). This finding corresponds with experimental evidence indicating THC exerts dose-dependent inhibition of osteoblast activity and bone resorption while suppressing alkaline phosphatase activity in osteoblast lineage cells, processes essential for bone formation and healing.^19^ These results corroborate emerging literature across arthroplasty, spinal fusion, and orthopaedic trauma domains, suggesting cannabis-associated bone-healing impairment comparable to established tobacco-related risks.^27,28^ Given osseous union constitutes a pivotal determinant of successful fracture healing, the heightened nonunion incidence in cannabis users represents a clinically consequential outcome that frequently necessitates revision surgery, thereby compounding patient morbidity and health care resource use.

Consequently, reoperation rates were significantly elevated among cannabis users, demonstrating a 117% increased risk (RR = 2.168, *P* < .001). This outcome could be directly linked to the cumulative complication burden described, wherein infectious sequelae, wound healing impairment, and nonunion often collectively necessitate reoperation. The observed temporal cascade of complications underscores cannabis use as a modifier of sustained surgical vulnerability beyond the immediate postoperative phase. This risk trajectory progresses from early wound compromise and infection to chronic hardware issues and structural failure, ultimately culminating in additional surgical interventions.

Given the emerging evidence linking preoperative cannabis use to increased risks of various adverse postoperative outcomes^5-7,16,21,25,29^ in conjunction with the clinical implications of our findings, cannabis use should be recognized as a modifiable perioperative risk factor, comparable to smoking and tobacco use. Our results build on and extend the only prior published study on this topic in ankle fracture ORIF by Dhodapkar et al,^5^ which found no significant increase in complications among cannabis-only users. However, their smaller sample size, inclusion of combined tobacco/cannabis groups, and limitation to 90-day outcomes may have reduced power to detect long-term surgical risks. In contrast, our larger, cannabis-specific cohort and long-term follow-up revealed significantly increased risks of nonunion and reoperation, aligning with trends in broader orthopaedic literature. Routine screening for cannabis use could be integrated into preoperative workflows, and patients should receive targeted counseling regarding its potential impacts on surgical outcomes. Moreover, patients may benefit from individualized perioperative management strategies, potentially encompassing enhanced postoperative wound surveillance, cessation of cannabis use, cautious surgical timing, or tailored postoperative care pathways that could help mitigate the elevated complication risk among cannabis users.

### Limitations

While this study provides valuable insights, it carries several inherent limitations. Its retrospective design precludes causal inference, allowing only associations to be observed. Cannabis use was identified through *ICD-9*/*10* codes and clinician documentation within electronic health records, which may introduce misclassification because of underreporting, inconsistent screening, or variability in clinical documentation. Although we strengthened specificity by requiring 2 distinct preoperative cannabis use diagnoses, this approach lacks detail on dosing frequency, duration, route of administration, and cannabinoid composition, limiting the ability to evaluate dose-response relationships. Additionally, undocumented cannabis use among controls may have attenuated observed associations. Similarly, although tobacco use was one of the variables incorporated in propensity score matching, limitations present with the data available in TriNetX do not allow us to quantify the extent of tobacco use. We were unable to account for fracture severity (eg, open vs closed) because of inconsistent coding across institutions. Although we matched for several comorbidities associated with healing risk, unmeasured differences in injury characteristics may still contribute to residual confounding. Our cohort’s relatively young mean age (~37 years) may limit generalizability to older populations. Outcome definitions were based on widely used diagnosis and procedure codes in orthopaedic research, but these codes have not been independently validated within the TriNetX network. Furthermore, TriNetX follow-up is encounter-based; patients without subsequent visits are assumed not to have experienced the outcome, potentially leading to undercapture of long-term events. For transparency, all codes used for outcome definitions are included in Appendix A. Lastly, variation in surgical technique, implant selection, and perioperative protocols across institutions may introduce additional unmeasured confounding and limit external validity. Future prospective studies should incorporate granular cannabis exposure data, validate abstinence status, and evaluate perioperative cessation strategies to clarify causal relationships and optimize patient care.

## Conclusions

This study found preoperative cannabis use to be associated with an increased risk for complications following ORIF for rotational ankle fractures, most notably postoperative infection, nonunion, and reoperation. These associations support recognizing cannabis use as a potentially modifiable perioperative risk factor analogous to tobacco use. Gaining deeper insight into the specific risks and underlying mechanisms linked to cannabis use will be crucial for enhancing perioperative risk assessment, improving patient counseling, and developing targeted strategies to optimize surgical outcomes following ankle ORIF.

## Supplemental Material

sj-pdf-1-fai-10.1177_10711007251385971 – Supplemental material for Preoperative Cannabis Use and Ankle ORIF Outcomes: Higher Risks of Infection, Nonunion, and Reoperation Supplemental material, sj-pdf-1-fai-10.1177_10711007251385971 for Preoperative Cannabis Use and Ankle ORIF Outcomes: Higher Risks of Infection, Nonunion, and Reoperation by Sri Tummala, Brandon A. Wood, Mehul M. Mittal, Senthil N. Sambandam and Dane K. Wukich in Foot & Ankle International
